# The 2024 Virtual Care Symposium “From Novelty to Sustainability”

**DOI:** 10.1089/tmr.2024.0076

**Published:** 2024-12-11

**Authors:** Elizabeth A. Krupinski

**Affiliations:** Department of Radiology & Imaging Sciences, Emory University, Atlanta, Georgia, USA.

Since 2020, a unique conference gathering experts in virtual care has taken place, each focusing on a different and timely topic. On March 13, 2024, the “From Novelty to Sustainability: How to Embed Virtual Care Into the Post-Pandemic Healthcare Delivery Template” Virtual Care Symposium, the fourth symposium in the series, was convened. It was hosted by Yale School of Medicine, Emory University, the University of Washington, and the Mayo Clinic Scottsdale. The symposium showcased initiatives in AI-driven virtual care, strategies for securing a return on investment (ROI) in digital health, and the role of libraries in expanding telehealth access.

As the title indicates, the symposium’s overall focus was around sustainability and trying to understand, now that the COVID-19 pandemic has receded, how we need to think about permanent transformation and not letting some of the amazing gains that we experienced during the pandemic simply revert to business as usual or even worse.

The symposium centered on three core topics with presentations from the experts followed by opportunities for audience comments and questions. The first segment focused on the integration of advanced technologies in virtual care and the potential for improving patient outcomes and health care efficiency. The speakers highlight the importance of virtual care in a post-pandemic health care system, emphasizing the need for clinical workflow redesign, robust IT infrastructure, and patient engagement. They discussed the role of AI, particularly RPA, ML, and GenAI in automating tasks, providing decision support, and improving patient care. Some examples of innovative programs included Emory's AI Ambient Listening program, which uses AI to generate clinical notes, improving patient and provider experience and wellness; and Houston Methodist's efforts in creating a “Smart Hospital,” including TeleNursing and connected care models, to enhance care delivery, improve outcomes, and reduce costs. The talks highlighted the fact that integration of these advanced technologies, while requiring significant investment and attention to integration, has the potential to transform health care delivery and improve both patient outcomes and health care efficiency.

The second segment was rather novel in the way it took place, having a discussion with panelists from different perspectives. They were given realistic pitches on tools/products for virtual care and asked to discuss how to move the field ahead and start evaluating ROI between leaders within health systems, payers, investors, and venture capitalists. The speakers were asked to focus on how to evaluate the ROI of digital health tools in a way that goes beyond simply considering their “innovation” or “coolness.” Panelists argued that health systems should evaluate digital health tools in the same way that they would evaluate any other device being introduced into the health system, focusing on factors such as whether the tool helps solve an institutional problem, the payment model in which it can be used, how it fits into pre-existing workflows, whether patients and providers will engage with it, and its actual ROI. They also discuss the importance of partnerships between health systems and digital health companies and ways partnerships can be structured. The importance of the timing of implementation to align with budget cycles and benefit design changes was also noted. Finally, there needs to be a clear strategic plan that guides the adoption of digital health tools and ensures that they are used to support the overall goals of the health system.

Finally, libraries are becoming essential telehealth hubs, particularly in underserved and rural communities, due to their accessibility, technology resources, and community trust. They bridge the digital divide by providing internet access, devices, and technical support to those lacking these resources at home. Libraries offer private spaces for telehealth appointments, ensuring confidentiality and a quiet environment. Libraries are well-distributed geographically, often surpassing hospitals in number and reach, especially in rural areas. This accessibility mitigates barriers to health care, such as transportation challenges and geographical distance. Libraries also serve as trusted intermediaries between health care providers and communities, hosting health education programs and partnering with local organizations to promote telehealth services. Successful implementation of telehealth in libraries necessitates infrastructure planning, staff training, and community engagement, highlighting their adaptability in meeting diverse health needs. Examples from Delaware, Utah, and Texas demonstrate successful library telehealth programs.

The three articles resulting from this symposium summarize these three core topic areas and collectively paint a picture of a health care landscape rapidly evolving to embrace virtual care. AI-driven innovations, sound investment strategies, and the crucial role of libraries as telehealth hubs are key themes that emerged from the “From Novelty to Sustainability” Virtual Care Symposium. The articles offer valuable insights into the challenges and opportunities associated with integrating virtual care into the post-pandemic health care delivery model.

